# Autonomic Cardiovascular Alterations in Chronic Kidney Disease: Effects of Dialysis, Kidney Transplantation, and Renal Denervation

**DOI:** 10.1007/s11906-021-01129-6

**Published:** 2021-02-13

**Authors:** Fosca Quarti-Trevano, Gino Seravalle, Raffaella Dell’Oro, Giuseppe Mancia, Guido Grassi

**Affiliations:** 1 Clinica Medica, Department of Medicine and Surgery, University Milano-Bicocca, Via Pergolesi 33, 20052 Monza, Italy; 2 Policlinico di Monza and University Milano-Bicocca, Milan, Italy

**Keywords:** Sympathetic nervous system, Baroreflex, Cardiopulmonary reflex, Renal failure, Hemodialysis, Renal transplantation, Renal denervation, Prognosis

## Abstract

**Purpose of Review:**

To review the results of studies of the effects of dialysis and kidney transplantation on the autonomic nervous system alterations that occur in chronic kidney disease.

**Recent Findings:**

Vagal control of the heart mediated by arterial baroreceptors is altered early in the course of the renal disease. Sympathetic activation occurs, with increases in resting heart rate, venous plasma norepinephrine levels, muscle sympathetic nerve traffic, and other indirect indices of adrenergic drive. The magnitude of the changes reflects the clinical severity of the kidney disease. Both the sympathetic and parasympathetic alterations have a reflex origin, depending on the impairment in baroreflex and cardiopulmonary reflex control of the cardiovascular system. These alterations are partially reversed during acute hemodialysis, but the responses are variable depending on the specific type of dialytic treatment that is employed. Renal transplantation improves reflex cardiovascular control, resulting in sympathoinhibition following renal transplantation if the native kidneys are removed. Sympathoinhibitory effects have been also reported in renal failure patients after bilateral renal denervation.

**Summary:**

Assessment of autonomic nervous system responses to dialysis and renal transplantation provides information of clinical interest, given the evidence that autonomic alterations are involved in the development and progression of cardiovascular complications, as well as in the prognosis of chronic kidney disease.

## Introduction

Autonomic control of the circulation undergoes profound alterations in chronic kidney disease, with important effects on the parasympathetic and sympathetic regulation of the heart and the peripheral circulation [1•, 2•]. These alterations may have important adverse clinical outcomes in patients with chronic kidney disease, resulting in both the occurrence of acute intradialytic hypotensive episodes and the development and progression of cardiovascular complications, such as hypertensive heart disease, coronary artery disease, heart failure, and major cardiac arrhythmias, leading to an increased risk of fatal and non-fatal cardiovascular events [1•, 2•]. Whether and to what extent these autonomic alterations are irreversible or can be favorably affected by hemodialysis and kidney transplantation is debated.

We begin this review by describing alterations in sympathetic and parasympathetic cardiovascular control that occur in the presence of chronic kidney disease and discussing the results of the studies performed by our group and others on this issue. We then describe the mechanisms responsible for these alterations, focusing in particular on the impaired reflex modulation of vagal and adrenergic cardiovascular drive. This is followed by an in-depth evaluation of a debated issue, the reversibility of autonomic dysfunction by the hemodialysis procedure, with specific emphasis on the data collected by our group. The modifications in autonomic cardiovascular regulation exerted by renal transplantation will be then discussed. Finally, emphasis will be given to the autonomic impact of renal denervation when used in the treatment of chronic kidney disease-related resistant hypertension.

## Autonomic Cardiovascular Alterations in Chronic Renal Disease

The first evidence that chronic kidney disease is characterized by cardiovascular autonomic dysfunction dates back 50 years, when Goldberger and coworkers and Soriano and collegue [3, 4] reported that the heart rate responses to the Valsalva maneuver, physiologically characterized by an increase (strain phase) followed by a reduction (release phase), undergo profound changes in uremic patients. Specifically, the heart rate lowering responses appeared to be compromised early in the clinical course of the kidney disease. These findings, which have been confirmed and expanded in the following years by other investigators [5, 6], were ascribed to an impairment in the parasympathetic regulation of sinus node activity [1•, 7, 8]. The alterations were suggested to be responsible for the inability of cardiac output to increase in response to the reduction in peripheral vascular resistance, leading to the frequent occurrence of intra- or post-dialytic hypotension [9].

Along with the parasympathetic alterations noted above, chronic kidney disease is also characterized by profound abnormalities in sympathetic cardiovascular control. These were originally described based on measurement of venous plasma norepinephrine concentrations [10–12]. This approach has the limitation that it is not possible to determine whether and to what extent the increased circulating levels of the adrenergic neurotransmitter reflect a true augmentation of sympathetic cardiovascular outflow. Increased plasma norepinephrine levels may depend not only on augmented secretion from adrenergic nerve terminals but also on reduced tissue clearance and/or impaired neuronal reuptake [13•]. Despite these limitations, assessment of sympathetic function in chronic kidney disease has been based for many years on the assay of venous plasma norepinephrine, which has been shown to be consistently increased, particularly in the more advanced stages of chronic kidney disease.

During the past 30 years, evaluation of human adrenergic cardiovascular drive has received renewed interest from investigators and clinicians due to the availability of new analytic techniques that can overcome the limitations of the plasma norepinephrine assay. These include the radiolabeled norepinephrine spillover technique, power spectral analysis of the heart rate signal, neuroimaging, and direct microneurographic recording of efferent postganglionic sympathetic nerve traffic in peripheral (brachial or peroneal) nerves [13•]. The norepinephrine spillover technique, which is based on the use of radiolabeled material, is largely employed in other clinical conditions characterized by sympathetic overdrive, such as heart failure, hypertension, obesity, and metabolic syndrome, for defining the patterns of regional adrenergic drive in the renal, coronary, and cerebral circulations. Its use in the patients with chronic kidney disease is both potentially dangerous due to the possibility of accumulation of radioactive tracers and of very limited value for understanding regional sympathetic function because the whole body clearance of radiolabeled norepinephrine depends on preserved renal function [13•].

Another approach to the investigation of autonomic function is the power spectral analysis of heart rate variability [13•]. This approach has appeal because it is noninvasive and relatively easy and inexpensive to perform. It also has significant limitations, particularly as a quantitative and specific indicator of cardiac sympathetic, as opposed to parasympathetic activity, and its insight does not extend beyond sympathetic control of heart rate [13•]. Despite these limitations, analysis of heart rate variability in uremic patients has confirmed that parasympathetic regulation of sinus node activity is impaired in uremic patients [14–17].

**Fig. 1:**
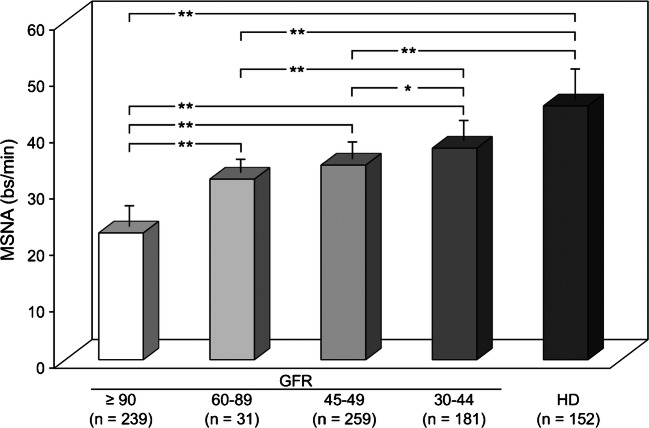
Muscle sympathetic nerve traffic (MSNA) values in different groups of patients subdivided according to the values of estimated glomerular filtration rate (GFR) or presence of hemodialytic procedure (HD). Numbers in parentheses refer to the patients belonging to each group. Data are shown as means ± SEM. Asterisks refer to the statistical significance between groups (*P<0.05,**P<0.01). Figure withdrawn from data of the meta-analysis of 29 microneurographic studies (Ref [18••])

In contrast, direct recording of efferent postganglionic muscle sympathetic nerve traffic provides direct information on the behavior of central sympathetic outflow in chronic kidney disease [13•]. These data were recently reviewed by our group in a meta-analysis of 29 studies with a total of more than 600 uremic patients participating [18••]. Our analysis revealed five major findings. First, similar to hypertension, heart failure, and obesity, chronic kidney disease is characterized by sympathetic nervous system activation which involves the heart and the peripheral circulation [13•, 19–23]. Second, the increase in sympathetic nerve traffic occurs in both mild-to-moderate and severe chronic kidney disease, indicating that sympathetic activation begins early in the clinical course of kidney disease and increases with the severity of renal impairment [20, 23]. Third, as has been described in hypertension and heart failure, the adrenergic overdrive occurring in the earlier phases of chronic kidney disease may play a compensatory role that preserves adequate tissue perfusion, but may cause over time adverse effects resulting in the development and progression of organ damage, such as left ventricular hypertrophy and diastolic dysfunction, reduction in arterial compliance, and impaired endothelial function and vascular distensibility [1•, 2•, 13•, 24–26]. Fourth, the magnitude of the adrenergic overdrive increases progressively as glomerular filtration rate falls in the different clinical phases of the disease (Fig. 1). This can be detected in chronic kidney disease of various etiologies, such as nephrosclerosis, chronic glomerulonephritis, and interstitial nephritis [19–23, 27]. Finally, the sympathetic overdrive does not occur at the level of the cutaneous circulation, presumably because of the adrenergic drive in the vascular bed of the skin [2•].

Another approach to assess sympathetic function is the neuroimaging technique, which makes use of very small amount of radiolabeled sympathetic amine (^123^metaiodobenzoguanidine) to image the sympathetic innervation of a given organ, in particular the heart [13•]. In uremic patients, there is rapid washout of the radiolabeled material from the heart, presumably due to reduced vesicular storage or to increased release of endogenous norepinephrine from cardiac adrenergic nerves [28, 29].

The easiest way to assess sympathetically mediated cardiovascular function is by evaluation of the resting heart rate. This is based on the evidence that elevated heart rate values (1) depend on increased adrenergic drive to the heart and, to a lesser extent, on reduced parasympathetic tone [1•], (2) are common in conditions characterized by sympathetic activation, such as heart failure, hypertension, and obesity [30], and (3) are directly and significantly related to well-established adrenergic markers, such as plasma norepinephrine and muscle sympathetic nerve traffic, in various forms of metabolic and cardiovascular disease, as well as chronic kidney disease [31]. However, a recent study by our group suggests that in chronic kidney disease, heart rate does not reflect the extent of functional impairment, based on the measurement of the estimated glomerular filtration rate [32]. This variance is from what is seen by measuring directly muscle sympathetic nerve traffic via clinical microneurography in patients with chronic kidney disease. This suggests that the sensitivity of heart rate as a sympathetic marker is lower in chronic kidney disease than in heart failure, obesity, or hypertension.

### Alterations in Reflex Cardiovascular Control

There is conclusive evidence that the impaired vagal control of heart rate and the enhanced sympathetic drive seen in chronic kidney disease have a reflex origin. The evidence for impaired vagal control of heart rate dates back to the observation that the bradycardic response to arterial baroreceptor stimulation via intravenous bolus injection of phenylephrine was significantly reduced in patients on chronic hemodialysis compared to age-matched healthy controls [7]. This finding was later confirmed using other methods of assessing sympathetic function, such as the bolus intravenous injection of angiotensin or inhalation of amyl nitrate, or evaluating spontaneous baroreflex sensitivity via power spectral analysis of the heart rate signal with the fast Fourier transform method [8, 14–17].

**Fig. 2:**
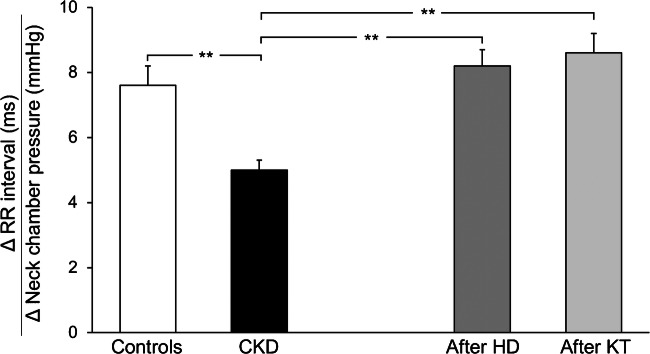
Baroreflex sensitivity values, derived from the ratio between R-R interval and neck chamber pressure changes, in control healthy subjects (Controls, *n*=10), in patients with chronic kidney disease (CKD, *n*=25), and in patients after hemodialysis (HD, *n*=25) or kidney transplantation (KT, *n*=9). Data are shown as means ± SEM. Asterisks refer to the statistical significance between groups (**P<0.01). Figure withdrawn from data of Reference [33]

Our group investigated autonomic reflex cardiovascular control in 25 young (age: 31.2±2.6 years, mean ± SEM) uremic patients maintained on hemodialysis three times a week over an average of 4 years of follow-up (34). We evaluated baroreceptor and cardiopulmonary receptor control of the circulation before and after hemodialysis in all patients and in a selected group of patients, following kidney transplantation [1•, 33, 34]. The study was approved by the Ethic Committees of the institutions involved. Carotid baroreceptor control of heart rate was assessed via the neck chamber technique [33], which allowed selective stimulation of carotid baroreceptors by progressively increasing carotid transmural pressure through application of negative pressures in the neck chamber. The reflex lowering of heart rate was measured over the 2–3 cardiac cycles immediately following the baroreceptor stimulus by analyzing the EKG tracing. Baroreflex sensitivity was expressed as the slope of the linear regression between lengthening of the R-R interval on the EKG and the negative pressure applied around the neck. As shown in Fig. 2, the sensitivity of the baroreflex was significantly reduced in uremic patients (black bars) compared to a group of 10 healthy age-matched controls (white bars), confirming the impairment in carotid baroreceptor control of the heart rate.

**Fig. 3:**
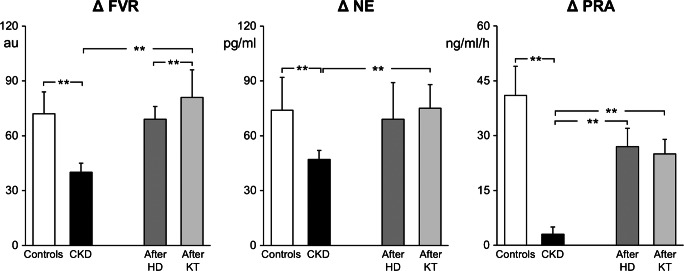
Percent reflex changes in forearm vascular resistance (FVR), plasma norepinephrine (NE), and plasma renin activity (PRA) in response to cardiopulmonary receptor deactivation in the different groups of patients of Fig. 2. Data are shown as means ± SEM. Other symbols and abbreviations as in Fig. 2. Asterisks refer to the statistical significance between groups (**P<0.01). Figure withdrawn from data of Reference [33]

We also evaluated cardiopulmonary receptor control of the forearm vascular resistance, venous plasma norepinephrine concentration, and plasma renin activity in these patients. We used the classic lower body negative pressure technique, which, by reducing venous return to the heart, deactivates the volume-sensitive receptors in the cardiac chambers and the pulmonary vascular bed [34]. As shown in Fig. 3 (white bars), this maneuver induced a marked increase in sympathetic vasoconstrictor tone in the skeletal muscle vascular bed, with significant increases in forearm vascular resistance and venous plasma norepinephrine concentration and renin activity in normal control subjects. All of these reflex responses were markedly attenuated in patients with chronic kidney disease, particularly in those with end-stage disease (black bars) [33]. Our more recent findings also indicate that, along with the arterial baroreflex and cardiopulmonary reflex alterations, chronic kidney disease is also characterized by marked tonic chemoreceptor activation, which may make an additional contribution to the increased sympathetic drive seen in these patients [35].

### Autonomic and Reflex Effects of Hemodialysis

Many studies have examined the impact of long-term hemodialysis on autonomic cardiovascular control [1•, 5, 19, 33, 36, 37]. While the results have generally failed to demonstrate significant improvement in the uremia-related autonomic dysfunction, some exceptions have been reported. As mentioned above, we evaluated carotid and cardiopulmonary reflex responses in uremic patients before and after acute hemodialysis procedure [33]. We found that, after a single hemodialysis session, carotid baroreceptor control of heart rate was significantly potentiated, and the vascular and humoral responses to cardiopulmonary receptor deactivation were significantly improved (Fig. 3). Among the factors proposed to explain the differing results reported in the various published studies, the leading one is the duration of the uremic state, which is associated with a neuropathy that is usually irreversible and unresponsive to therapeutic interventions such as hemodialysis. In addition, the type of dialytic procedure employed may affect autonomic responses. This may be the case for nocturnal hemodialysis, which has been reported to reduce plasma norepinephrine levels, enhance endothelium-dependent vasodilatation, improve baroreflex sensitivity, and normalize blood pressure of hypertensive patients with end-stage renal disease [38, 39]. This may be also the case for hemofiltration or ultrafiltration, but not for peritoneal dialysis, which has not been reported to alter the autonomic derangements detected in uremic patients [1•, 36, 40]. Use of frequent (daily) short-term hemodialysis sessions without increasing total dialysis time has been shown to significantly reduce sympathetic nerve traffic, thereby creating a valid therapeutic option to the usual three times a week procedure [41].

### Autonomic and Reflex Effects of Kidney Transplantation

The evidence reported above does not clarify a crucial issue, whether and to what extent the autonomic and reflex abnormalities that occur in chronic kidney disease have a functional rather than a structural nature and whether they can be reversed by treatment. This question received a clearcut answer from the results of the studies that assessed the potential impact of kidney transplantation on autonomic and reflex function. These studies showed that parasympathetic control of the heart clearly improves after renal transplantation. This was documented years ago by evaluating heart rate responses to the Valsalva maneuver and the expiration/inspiration ratio and more recently via the power spectral analysis of the heart rate signal [1•, 5, 6, 15, 42]. Baroreflex control of heart rate, as assessed by the neck chamber technique, was also significantly improved in the 9 uremic patients that we examined 3 months after kidney transplantation (Fig. 2) [33]. Similar potentiation was has been detected by other investigators using the vasoactive drug infusion technique [1•].

Similarly to the arterial baroreflex, the cardiopulmonary reflex undergoes significant improvement after kidney transplantation. We found that the increase in forearm vascular resistance, venous plasma norepinephrine, and plasma renin activity triggered by the cardiopulmonary receptor deactivation induced by a mild degree of lower body negative pressure was significantly potentiated after kidney transplantation, the reflex responses becoming almost indistinguishable from those seen in heathy persons [33].

Using venous plasma norepinephrine as marker of adrenergic drive, we and others found a significant reduction following renal transplantation [1•, 2•, 33]. This was also the case when ^123^metaiodobenzoguanidine imaging was employed [42]. However, other methods of assessing sympathetic function did not support the neuroadrenergic deactivation seen following renal transplantation with these methods. This was particularly true for the results of the studies based on clinical microneurography, which failed to demonstrate significant sympathoinhibitory effects of renal transplantation [20]. This finding is likely related to the sympathoexcitation elicited by cyclosporine, tacrolimus, or other immunosuppressive agents administered to avoid kidney allograft rejection [43, 44]. Retention of the diseased native kidneys is another cause of sympathoexcitation, and surgical removal of the native kidneys combined with renal transplantation may permit almost complete normalization of sympathetic cardiovascular function [20]. This finding suggests that signals arising from the native kidney(s) that activate the central nervous system may mask the true sympathoinhibitory effects of kidney transplantation in uremic patients [45].

### Effects of Renal Denervation on Renal Failure-Related Autonomic Alterations

Recent clinical studies have shown that bilateral ablation of the renal nerves may result in sustained benefits for renal function in patients with chronic kidney disease [46•, 47]. Marked reductions in muscle sympathetic nerve traffic and whole-body norepinephrine release have also been seen in this setting [22]. No information is available regarding the impact of renal denervation on the parasympathetic cardiovascular control, although the observation that no clearcut heart rate changes were observed after renal denervation [22, 46•, 47, 48] speaks against a major effect of the procedure on the vagal regulation of the heart. Similarly, no observation has been reported on the impact of renal denervation on reflex cardiovascular control in patients with chronic kidney disease, although an improvement in baroreflex control of sympathetic nerve traffic has been reported in patients with true resistant hypertension and preserved renal function [49].

## Conclusions

This review raises a question should be advanced, namely, why we should assess and define the autonomic cardiovascular profile in patients with chronic kidney disease. The answer is based on evidence that autonomic function has independent prognostic relevance in these patients. This has been shown for venous plasma norepinephrine, where elevated circulating levels of this adrenergic neurotransmitter (and thus likely greater levels of sympathetic activation) have been associated with lower survival rate, even after data were adjusted for confounders [50]. This has been also shown for abnormalities in 24-h heart rate power spectral analysis, greater levels of heart rate variability being associated with an increased risk of developing life-threatening cardiac arrhythmias, and sudden death in uremic patients [51, 52]. Thus, evaluation of the autonomic cardiovascular profiles of patients with chronic kidney disease provides useful information for the assessment of their individual cardiovascular risk and thus for defining the most appropriate therapeutic intervention.
